# Studies Regarding the Antimicrobial Behavior of Clotrimazole and Limonene

**DOI:** 10.3390/antibiotics11121816

**Published:** 2022-12-14

**Authors:** Verginica Schroder, Nicoleta Radu, Petruta Calina Cornea, Oana Andreia Coman, Lucia Camelia Pirvu, Mohammed Shaymaa Omar Mohammed, Amalia Stefaniu, Lucia Pintilie, Marinela Bostan, Mihai Dan Caramihai, Viviana Roman

**Affiliations:** 1 Faculty of Pharmacy, University Ovidius of Constanta, 900527 Constanta, Romania; 2 Faculty of Biotechnology, University of Agronomic Sciences and Veterinary Medicine of Bucharest, 011464 Bucharest, Romania; 3 Department of Biotechnology, National Institute of Chemistry and Petrochemistry R&D of Bucharest, 060021 Bucharest, Romania; 4 Faculty of Medicine, University of Medicine and Pharmacy Carol Davila of Bucharest, 020021 Bucharest, Romania; 5 Department of Pharmaceutical Biotechnology, National Institute of Chemical Pharmaceutical R&D of Bucharest, 031299 Bucharest, Romania; 6 Department of Immunology, National Institute of Pathology and Biomedical Sciences R&D “Victor Babeș’’, 050096 Bucharest, Romania; 7 Center of Immunology, Institute of Virology Stefan S. Nicolau, 030304 Bucharest, Romania; 8 Faculty of Computer Sciences, Politehnica University of Bucharest, 060042 Bucharest, Romania

**Keywords:** clotrimazole, limonene, synergistic effect

## Abstract

The paper presents the results of the studies performed to establish the effect of the mixtures between limonene and clotrimazole against microbial pathogens involved in dermatological diseases, such as *Candida albicans*, *Staphyloccocus aureus*, and *Escherichia coli*. Preliminary data obtained from the studies performed in microplates revealed a possible synergism between the mixture of clotrimazole and limonene for *Staphylococcus aureus*. Studies performed “in silico” with programs such as CLC Drug Discovery Workbench and MOLEGRO Virtual Docker, gave favorable scores for docking each compound on a specific binding site for each microorganism. The tests performed for validation, with the clotrimazole (0.1%) and different sources of limonene (1.9% citrus essential oils), showed a synergistic effect on *Staphylococcus aureus* in the case of the mixtures between clotrimazole and the essential oils of *Citrus reticulata* or *Citrus paradisi*. The studies performed on *Staphylococcus aureus* MRSA showed a synergistic effect between clotrimazole and the essential oils obtained from *Citrus bergamia*, *Citrus aurantium*, or *Citrus paradisi*. In the case of *Pseudomonas aeruginosa*, essential oils and clotrimazole used alone did not exhibit antimicrobial activities, but the mixtures between clotrimazole and the essential oils of *Citrus bergamia* or *Citrus sinensis* exhibited a synergistic antimicrobial effect.

## 1. Introduction

Citrus fruits generate large amounts of residues, generally made up of peels, seeds, and membranes. The amount of this waste exceeds 120 million tons annually worldwide. The recovery of these residues can be completed to obtain raw materials for the pharmaceutical, food, or biofuel industry [1]. Citrus peels contain essential oils, polyphenolic compounds, and carotenoids, the use of which has been recognized as safe by the U.S. Food and Drug Administration [1,2]. The most important products obtained from citrus peels are essential oils (EO). The EO content in limonene (1-methyl-4-(1-methyl ethenyl)-cyclohexene) can reach 97–98%. Limonene can be separated from citrus peels by microwave-assisted extraction (in the absence of solvents) or by extraction with supercritical fluids [2]. In medicine, citrus oil is important in treating bacterial infections with *E. coli* and *S. aureus* due to their content of limonene, β-citronellol, carvacrol carvone, eugenol, and trans-cinnamaldehyde [3,4,5,6]. Han et al. mentioned that limonene has no activity on *E. coli* but inhibits the development of * S. aureus*, evaluating a value for MIC (minimum inhibitory concentration) and MBC (minimum bactericidal concentration) of 21 μg/mL [7]. The mechanism of action is based on the destruction of cell membrane integrity and the reduction of metabolic activity at c = 20 μg/mL [8]. Li and collab. reported that the essential oil obtained from *Citrus medica* var. *Sarcodactylis*, which contains about 45% limonene, inhibits the growth of *S. aureus* and *E. coli*, with the obtained inhibition diameters being 19 mm for *S. aureus* and 11.2 mm for *E. coli*, respectively. The minimum inhibitory concentration and the minimum bactericidal concentration MBC for the two microorganisms were: MIC= 0.625 mg/mL and MBC = 1.25 mg/mL for *S. aureus* and MIC = MBC = 1.25 mg/mL for *E. coli*, respectively. Monitoring the growth over time of those two microorganisms, whose culture medium was supplemented with this type of EO, showed that in the case of *E. coli* the number of colony-forming units (CFU) decreases by about 2.5 logarithmic units after 4 h of exposure, while for *S. aureus* the CFU number decreases by 3 logarithmic units after 2 h of exposure, at an EO concentration corresponding to the MIC value. The destruction of microbial cells is achieved due to the permeability of cell membranes under the influence of components from EO, a process followed by the loss of cell integrity and cell death [9]. Other researchers have found that for fungi from the *Candida* genus, mixtures of essential oils and antibiotics such as clotrimazole (CT) show synergies [10]. Studies performed by Nidhi et al. with an essential oil obtained from the leaves of *Citrus aurantium* (EOCA) showed that the mixture between a solution of 10% essential oil in DMSO and a solution of antibiotics such as fluconazole (FCZ) or amphotericin (APH), which contains 20 μg/mL antibiotic in DMSO, has a synergistic effect on two strains of *C. albicans* (ATTC 90,028 and MTCC277). The values obtained for MIC in the case of individual components were found as follows: (0.15 ÷ 0.31)% EOCA, (0.15 ÷ 0.62)% FCZ and 0.62% AMP (ampicillin). If the components are mixed in the concentrations mentioned above, the MIC value decreases by one or two orders of magnitude. If the two components in the mixture (EOCA + antibiotic reagent) are introduced in the growth medium of the *C. albicans*, then the MIC value obtained for the mixture (EOCA + FCZ) is in the range of (0.018 ÷ 0.075)%. In the case of the mixture (EOCA + AMP), the MIC value is in the range of (0.0093 ÷ 0.075)% [11]. In their studies, Gupta and collab. have demonstrated that if the culture medium of *E. coli* or *S. aureus* is supplemented with soluble Ca^2+^ or Mg^2+^-based compounds, the CMI for limonene increases significantly (Gupta et al. 2021). Thus, in the case of *E. coli*, in the absence of Ca^2+^ or Mg^2+^ cations, MIC_limonene_ = 16 μg/mL; however, if the culture medium is supplemented with 10 mM Mg^2+^ or 10 mM Ca^2+^, then the MIC value increases twice (MIC = 32 μg/mL). In the case of *S. aureus*, supplementation of the culture medium with Ca^2+^ or Mg^2+^ at the same concentration (10 mM) leads to an increase in MIC values from 8 μg/mL to 1024 μg mL [8]. Lesgard et al. reported synergies in the use of combinations between essential oils and synthetic chemotherapeutics used in antitumor therapies [12]. Among the studied bioactive molecules with synergistic activity are compounds such as limonene, geraniol, carvacrol, α-humulene, and α-bisabolol. These compounds have antitumoral activity in leukemia, neuroblastoma, breast, liver, and skin cancer. In the case of skin tumors (melanoma), the components from EO activate the apoptosis processes by activating the proteins from the caspase’s family, with their presence signaling the beginning of the tumor cell destruction [12]. The same authors specify that limonene reduces the formation of blood vessels that supply nutrients to tumor formation, with the expression of VEGF (vascular endothelial growth factor) [13] being reduced by 60% [12]. Shojaei and collab. reported that the growth of tumor cells is inhibited in preclinical experiments performed with limonene on patients with various tumors including melanoma. The similarities are also observed in in vitro tests performed on tumor cell lines type U251, UACC-62, HT-29, MCF-7, NCI-H460, NCI/ADR/RES, OVCAR -03, and K562 [14]. In vitro tests performed on DU-145 tumor cell lines have shown that the mixture between an antitumor reagent (i.e., docetaxel) and limonene induces apoptosis and increases the generation of reactive oxygen species (ROS) [14,15]. In the case of K562 and HL60 cell lines, limonene acts at the level of mitochondria. Tests performed on lab animals with induced melanoma have shown that topical administration of limonene has stopped the tumor growth by inhibiting the prenylation process (i.e., inhibiting the isoprenylation process of small G proteins) [16,17]. Statistically, it has been found that an average of 8% of patients suffer from skin infections [18]: infections caused in 20% of cases of *S. aureus* MRSA. The treatments chosen in these cases include the administration of antibiotics such as oxacillin or anti-staphylococcal penicillin such as dicloxacillin and nafcillin [19]. Since 2002, the Infection Disease Society of America has issued an alert regarding the lack of antibiotics for infections with resistant microorganisms [18]. Hence, there is a need to develop new antibiotic delivery systems, a direction of great interest in the management of dermatological diseases [20], with most of them being caused by microorganisms included in the skin microbiome such as *S. aureus*, *S. epidermis*, *E. coli*, *P. mirabilis*, *P. aeruginosa*, or enterobacteria [21]. Silver-based nanoproducts and clotrimazole (CT), an imidazole derivative (1-[(2-Chlorophenyl) (diphenyl) methyl]-1H-imidazole), have been developed for antibiotic-resistant microorganisms such as *S. aureus* MRSA. This product decreases the minimum inhibitory concentration (MIC); in the case of *S. aureus*, the MIC decreases from 31.25 μg/mL for clotrimazole (CT) to 9.76 μg/mL for the Ag and CT-based nanoproduct. In the case of MRSA, MIC decreases from 31.25 μg/mL to 15.62 μg/mL [22]. Another approach to infections with resistant microorganisms is based on the use of microemulsions made from chitosan, CT, Tween 80, and propylene glycol [23]. The microemulsion result is effective in *C. albicans* infections for which a MIC = 10 μg/mL is obtained [23]. Clotrimazole shows antimicrobial activity for *S. aureus* MRSA, and in infections found in dogs with *S. pseudintermedius* and *S. intermedius*, for which CMI_50_ = CMI_90_ = 1 mg/mL [24]. Another strategy in the case of poorly soluble antibiotics such as CT is to incorporate them into ufosomes, using cholesterol and sodium oleate as reagents (ufosomes are vesicular systems made with unsaturated fatty acids) [20]. Hydroxypropyl methylcellulose is used to improve the dispersion index of this lipidic formulation [20]. Grimling et al. have improved the efficiency of CT by incorporating it into chitosan with a high molecular weight, by grinding the two components together (dry grinding), or by kneading these two components. The process consists of mixing chitosan and CT with ethyl alcohol until a consistent suspension is obtained, followed by the evaporation of the solvent at 30 °C [25]. The physical-chemical determinations performed by X-ray diffraction and infrared spectrometry have shown that there are no physicochemical interactions between the two components. Solubility tests have shown that the product obtained by dry grinding of the two components is the best variant/choice, with the latter acting synergistically against *Candida* sp., because the required dose of antibiotic decreases significantly in the presence of chitosan (MIC_chitosan_ > 500 mg/mL; MIC_CT_ < 31.25 mg/L; MIC_(chitosan+CT)_ < 7.8 mg mL). Regarding CT’s mechanism of action, it was demonstrated that it interferes with the biosynthesis of ergosterol, with the high concentrations of CT blocking its synthesis (ergosterol represents an essential constituent of the cytoplasmic membrane in fungi, being one of the targets of antifungal antibiotics) [25]. In a study performed on the cell lines initiated in a lab from chemotherapy-resistant leukemia cells taken from patients, Ito et al. have shown that the imidazoline derivative, called CT, inhibits their development [26], when these are exposed to an environment containing 10 μM CT. The mechanism of action consists of the depletion of Ca^2+^ deposits from tumor cells, a process followed by apoptosis. At the concentration of CT used in the experiment (10 μM CT), the normal cells are not affected. In vitro studies performed by Penser and Beitner on tumor cell lines type LL2 (Lewis Lung Carcinoma) and CT-26 (Colon Adenocarcinoma), with/using CT, showed that the derivative of Imidazole reduces the level of Adenosine triphosphate (ATP) in cells, with their proliferation being completely inhibited after 3 h of exposure at CT [27]. Benzaquen and collab. have reported similar antitumor effects, in studies performed in vitro on tumor cell lines type A549 (human lung adenocarcinoma), HT-29 (human colon adenocarcinoma), MM-RU (human melanoma), and B16-F1-36 (mice melanoma) [28]. Proapoptotic effects on melanoma tumor cell lines were and continue to be also reported by other researchers [29,30,31].

Despite the biological effects reported by the literature, clotrimazole’s main disadvantages are its low solubility in aqueous media, and the adverse effects it can generate. Thus, the dermal administration may cause itching, hives, and even sensitivity to this antibiotic. When administered orally, this compound may disturb the synthesis of mesenchymal liver enzymes; in the case of intraperitoneal (i.p.) administration, it may cause a heart attack. Regarding the toxicity, the tests conducted on lab animals (rats and mice) indicate for the case of a single ingestion, a value for DL50 = 708 mg/kg, and, respectively, of 761 mg/kg. In the case of i.p. administration, the DL50 = 445 mg/kg and, respectively, 108 mg/kg. For these reasons, the use of clotrimazole is limited at topical administration [32,33].

Taking into account the results reported in the literature, it is estimated that the essential oils obtained from citrus residues (containing mainly limonene) and the antibiotic reagent named CT present interest in the management of dermatological diseases, due to the broad spectrum of action of the two components and to the possibility of these to act synergistically, thus being important in the actual context of increased resistance to antibiotics of pathogenic microorganisms.

The main objective of the studies performed was to establish if the mixture between limonene (and different natural sources which contain limonene) and clotrimazole can exhibit a different behavior against the main microbial pathogens involved in dermatological diseases. This main objective was accomplished during three types of investigations, made in vitro, on microplates; in silico with the help of docking programs such as CLC Drug Discovery Workbench and MOLEGRO Virtual Docker; and finally again in vitro, on Petri plates, with natural sources of limonene such as the essential oils from different citrus species.

In the experiments performed in vitro, on microplates, the main purpose was to evaluate the antimicrobial susceptibility of limonene, clotrimazole, and the mixtures between them, for three microorganisms: *Candida albicans*, *Escherichia coli*, and *Staphylococcus aureus.* The aim of the tests performed “in silico” was to establish the chemical interactions between limonene and/or clotrimazole with each specific binding site from *Candida albicans*, *Escherichia coli*, and *Staphylococcus aureus*, using two programs of docking studies: CLC Drug Discovery Workbench and MOLEGRO Virtual Docker. In the third experimental part, we aimed to test “in vitro” the antimicrobial action of some natural sources of limonene (different essential oils from citrus species) and mixtures of each one with clotrimazole for *Candida albicans*, *Escherichia coli*, *Staphylococcus aureus*, *Staphylococcus aureus MRSA*, and *Pseudomonas aeruginosa*. These tests have been performed in order to validate “the proof of concept” resulting from studies made on microplates and from docking studies performed “in silico”. Finally, based on pieces of information obtained in the three types of studies, we will select the best natural sources of limonene, which can be used in pharmaceutical formulations with clotrimazole, with improved antimicrobial properties.

## 2. Materials and Methods

### 2.1. Reagents

Limonene (97%) was purchased from the local Merck Bucharest, Romania. Clotrimazole was used as a liquid solution with 10.87 mg CT/mL in polyethylene glycol (Biofarm, Bucharest, Romania).

Essential oils of *Citrus sinensis*, *Citrus limon*, *Citrus aurantium*, *Citrus paradisi*, *Citrus reticulata* (green and red), and *Citrus bergamia* were purchased from the Romanian market, and characterized by gas chromatography coupled with a mass spectrometer (GC-MS Table 1).

### 2.2. Microorganisms and Culture Media

Mueller Hinton broth (Oxoid) was used as a culture medium for *Escherichia coli* ATTC 11303, *Staphylococcus aureus* ATTC 25923, *Staphylococcus aureus* MRSA ATTC 33592, and *Pseudomonas aeruginosa* ATTC 1338. Potato dextrose broth (BD Difco Sigma Aldrich, Bucharest, Romania) was used as a liquid culture medium for *Candida albicans* ATTC 10231.

Experiments on Petri plates were conducted using fresh microorganisms (growth during 24 h for bacteria and 48 h in the case of *C. albicans*, growth on TSA (Tryptic soy agar, Sigma Aldrich. Bucharest, Romania), and PDA (potato dextrose agar, Sigma Aldrich, Bucharest, Romania)).

### 2.3. Devices

Optical density measurements were made on microplate reader type Dynatex at 600 nm (Dynex Technologies-MRS, Chantilly, VA, USA). The microplate was incubated at 37 °C (incubator type Cole Palmer H2200 Vernon Hills, IL, USA) in the case of bacteria, and, respectively, at 25 °C in the case of *C. albicans*. All experiments were made in laminar hood type Faster Bio 48, (Cornaredo, Italy).

A GC–MS/MS TRIPLE QUAD Agilent 7890 A (Santa Clara CA, USA) used in the analysis of the essential oil was equipped with a DB-WAX capillary column (30 m length, 0.25 mm internal diameter, 0.25 mm film thickness) and helium as the carrier gas at 1 mL/min. For the essential oils analysis, the oven temperature was initially set at 100 °C, held for 2 min, then increased gradually to 280 °C, with 4 °C per minute and 6 min holding time. The GC injector and MS ion source temperatures were 250 °C and 150 °C, respectively. The transfer line temperature was 280 °C. The MS detector was operated in EI mode at 70 eV, with an *m*/*z* scanning range of 50–450.

### 2.4. Experimental Design on Microplates

Solutions of limonene, CT, and a mixture of them, with concentrations in the range of (5000 ÷ 2.37) μg/mL, were made in Mueller Hinton broth and in potato dextrose broth, respectively, in 96-well plates. Each inoculum was made in a sterile physiological serum containing suspensions of *S. aureus*, *E. coli*, or *C. albicans*, prepared according to McFarland standard 0.5. The inoculation rate in each well was made using a volumetric report of 1:10. The optical density of each plate was measured by a plate reader device, without microorganism inoculum, and with microorganism inoculum, respectively, at the time 0. The plate was incubated for 24 h at 37 °C in the case of bacterial strains, and at 25 °C for 48 h in the case of fungal strains. After incubation, each plate’s optical density (OD) was measured. Optical density at 24 h was assigned by the difference between OD at 24 h and 0 h and culture media without microbial inoculants. Each experimental variant was made in 3 repetitions.

The results are presented as an average value, with standard deviations. The total viable cells (UFC) after 24 h and, respectively, after 48 h were determined by taking out 100 μL from each well, diluting it properly, and spreading it out on the Petri plates; with a solid specific culture media (Mueller Hinton agar for bacteria and, respectively, potato dextrose agar for fungi); incubating at 37 °C in the case of bacteria and, respectively, at 24 °C for 48 h for *C. albicans*; counting the total number of colonies formed and reporting for each case. Results were reported as a log of colonies forming units (logCFU).

### 2.5. Docking Studies

Predictions regarding potential interactions between the selected active molecules and selected targets were achieved by molecular docking studies using the CLC Drug Discovery Workbench ( QIAGEN, Aarhus, Denmark) and MOLEGRO Virtual Docker programs (Molexus IVS, Odder, Denmark), respectively. Both approaches considered the occurring interactions between the native (co-crystallized) ligands, as references, specific to each studied microorganism, in complex with the selected receptor protein, imported from the International Protein Database (PDB Bank, PDB consortium). The studies performed with CLC Drug Discovery Workbench (QIAGEN Aarhus, Silkeborgvej 2, Prismet 8000, Aarhus C, Denmark) have been accomplished according to the following docking protocol [34]: ligand and protein preparation, setup binding site, docking simulations for the co-crystallized ligand, validation, docking of the investigation ligands, and results validation.

The results were given in terms of the docking score and hydrogen bonds created with the amino acids’ residues from the amino acid group interaction within the active catalytic site. Thus, the obtained data have been used to predict the binding modes, the binding affinities, and the best orientation of the docked compounds in the active site of the protein receptor. Additionally, the molecular properties of the small molecules, such as parameters of Lipinski’s rule of five [35], i.e., the molecular weight, number of hydrogen bond donors, number of hydrogen bond acceptors, and Log P (octanol-water partition coefficient), have been calculated. The studies performed with MOLEGRO have been accomplished according to the following docking protocol: importing the protein receptor from the PDB bank, preparation of the protein receptor, detection of cavities, search space setup, and docking simulation. The docking score and hydrogen bonds created with the amino acid residues from the group of interactions have been used to assess the binding modes and the orientation of the docked compounds in the active site of the protein receptor. In both approaches, the protein–ligand complex has been realized based on the X-ray structure of *Candida albicans* Dihydrofolate *Reductase*, downloaded from the Protein Data Bank PDB ID: 1AI9 [36,37]; in the case of *S. aureus*, the protein–ligand complex has been realized based on the X-ray structure of Wild-type *Staphylococcus aureus* DHFR in complex with trimethoprim, downloaded from the Protein Data Bank (PDB ID: 2W9H) [36,38]. For *Escherichia coli*, the protein–ligand complex has been realized based on the X-ray structure of *Escherichia coli K12* (protein Alpha-ketoglutarate-dependent dioxygenase AlkB), downloaded from the Protein Data Bank, PDB ID: 4JHT [36,37,38,39]. The two simulation programs considered the same co-crystallized (native ligand) as a reference, respectively:

- in the case of *C. albicans*, the follow was considered as a ligand (co-crystallized): NADPH Dihydro-Nicotinamide-Adenine-Dinucleotide Phosphate (NDP);
- in the case of *S. aureus*, the following was considered as a ligand: Trimethoprim (TOP);
- in the case of *E. coli*, the following was considered as a ligand: 8-hydroxyquinoline-5-carboxylic acid (8XQ). The co-crystallized ligands introduced in the protein fragments chosen from the PDB bank also guided the docking simulations for the studied ligands (clotrimazole; limonene), in the same binding site. In the case of the selected protein fragments, the two programs validated the docking protocol, both for each co-crystallized (the reference ligands), and the selected ligands (limonene and clotrimazole).

During the simulations, the co-crystallized ligand was removed from the protein complex; in its place, the studied molecule was re-docked. Each program calculated the length of the detected hydrogen bonds and the docking score. The predictions were favorable to the initially assumed docking mechanism if values close to those of the natural ligand were obtained. Both programs detected the binding site and the binding pocket, complementary to a specific ligand.

### 2.6. Results Validation

**Figure 1:**
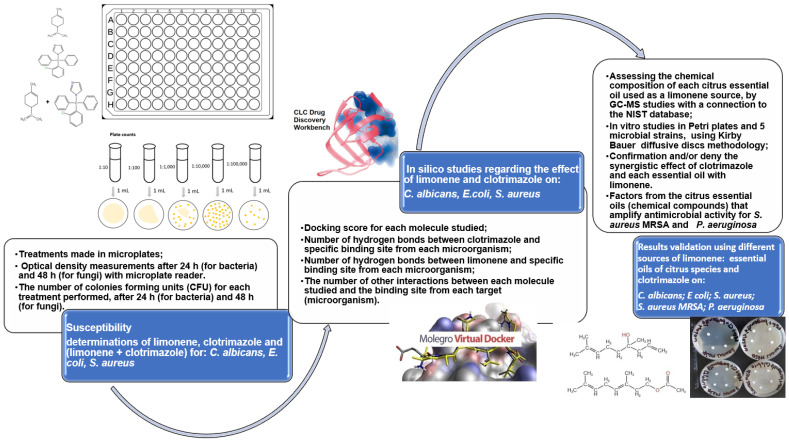
The methodology presented in studies regarding the antimicrobial behavior of clotrimazole and limonene.

Results validations were made on the Petri plates with Mueller Hinton culture media in the case of bacteria and, respectively, with PDA (potato dextrose agar) culture media in the case of fungi, using five microorganisms (*Candida albicans*, *Escherichia coli*, *Staphylococcus aureus*, *Staphylococcus aureus MRSA*, and *Pseudomonas aeruginosa*). The inoculum of each microorganism was prepared as a suspension in sterile saline solution (0.9% NaCl) using a fresh culture of each microorganism (fresh culture: 24-h-old bacteria and 48-h-old fungi), according to standard McFarland 0.5. The sterile inoculum of each microorganism was spread on the surface of each Petri plate with a cotton swab. After 20 min, in each Petri plate the cellulose discs with 0.6 mm diameter (3 or 5 cellulose discs for each treatment), impregnated with a solution that contained clotrimazole (0.1%, w/w), essential oils of citrus (1.9%), or a mixture of them at the same concentrations were added. All solutions were made with propylene glycol as a solvent. The Petri plates were incubated at 37 °C for 24 h (in the case of bacteria) and, respectively, for 48 h at 25 °C for *Candida albicans*. The results obtained are presented as the average diameter of inhibition with a standard deviation. The methodology used in the study is presented in Figure 1.

## 3. Results

### 3.1. Studies Performed on Microplates

#### 3.1.1. Studies on Microplates Performed on *E. coli*

Studies performed on *E. coli* exposed to limonene reveal that this compound inhibits cell growth at concentrations in the range of (5000 ÷ 39) μg/mL (Table 2 and Table 3). Due to this fact, at these concentrations, we considered that the studied microorganism is susceptible (S). At concentrations of (9.5 ÷ 2.37) μg/mL, the values of the optical densities (OD) are in the range of (0.05 ÷ 0.01) units, and we considered that, at these levels of limonene, the microorganism is intermediate (I) (or susceptible to increased exposure, according to EUCAST modification established in 2022 [40]). These observations are in agreement with results obtained by other researchers [3,8,9] regarding limonene activity against *E. coli.* In the case of exposure to *E. coli* at CT (Table 2 and Table 3), the measurements made after 24 h showed that, at concentrations of CT in the range of (5000 ÷ 19) μg/mL, the development of cells is inhibited, and the microorganisms are considered to be susceptible. For CT concentrations in the range of (9.5 ÷ 4.75) μg/mL, the OD range is (0.08 ÷ 0.06) units, and we considered that, at these CT levels, the microorganism has intermediate susceptibility at concentrations of CT less than 4.75 μg/mL and the microorganisms are considered resistant (R).

The results obtained are in agreement with those obtained by other scientists [41,42,43] who have reported similar biological activities of CT against *E. coli*. Under the influence of a mixture between limonene and CT (Table 1 and Table 2), the concentration at which *E. coli* is susceptible ranges from 5000 μg/mL to 9.5. μg/mL; at concentrations less than/below 9.5. μg/mL, microorganisms are resistant. In this case, the concentration range for which *E. coli* is susceptible is larger than the case of using only limonene or CT. These results permit us to affirm that by using a mixture between limonene and CT in equal parts, the antibiotic reagent concentration at which the microorganism is susceptible can be lowered to 4.75 μg/mL.

#### 3.1.2. Studies on Microplates Performed on *S. aureus*

**Figure 2:**
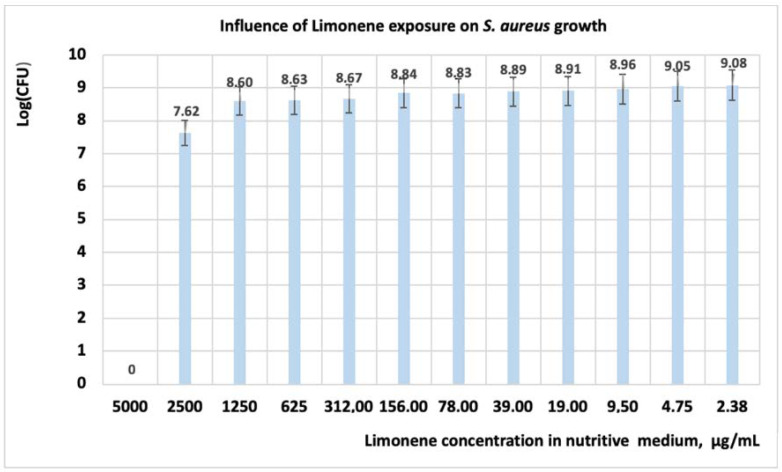
Influence of limonene exposure on *S. aureus* growth, after 24 h. At a concentration of limonene in the culture medium higher than 2500 μg/mL, *S. aureus* is inhibited.

Studies performed on *S. aureus* revealed that under the influence of limonene, the growth of the cells is inhibited at a concentration of 5000 μg/mL (Table 4, Figure 2), with the studied microorganism being susceptible at this value (Table 5).

When using 2500 μg/mL limonene, the OD value is 0.007, and at this value of concentration, the microorganism is considered with intermediate susceptibility. If the concentration of limonene in the culture medium is less than 2500 μg/mL, then the *S. aureus* cells are resistant. Regarding the number of CFU, its value decreases with one logarithmic unit to the concentration of 1250 μgL/mL (Figure 2); at the level of 2500 μg/mL, the CFU decreases with 1.5 log units, and at 5000 μgL/mL all cells are inactivated. Regarding the mechanism of action, studies performed with limonene on *S. aureus* revealed that this compound inhibits bacterial growth by the destruction of cell wall integrity [9].

**Figure 3:**
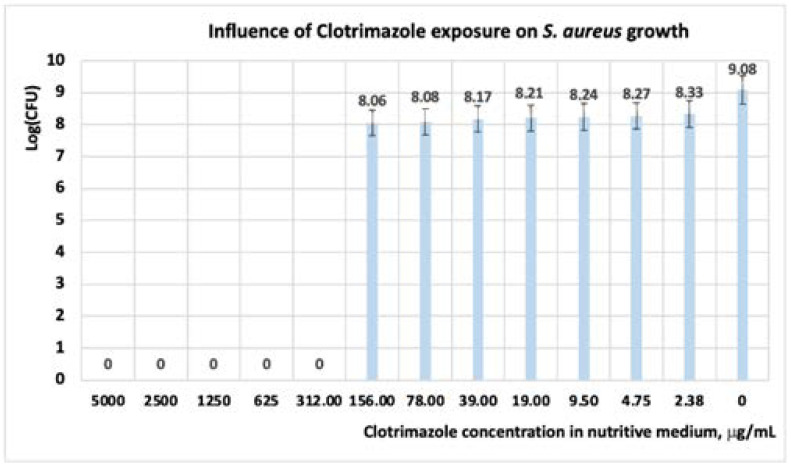
Influence of CT exposure on *S. aureus* growth, after 24 h. At a concentration of clotrimazole in the culture medium higher than 156 μg/mL, *S. aureus* is inhibited.

Following the exposure of *S. aureus* at CT the results showed that, at concentrations of CT in the range of (5000 ÷ 312) μg/mL, the development of cells is inhibited; hence, the microorganisms are susceptible. When concentrations of CT less than 312 μg/mL are used, the microorganism is resistant. Regarding the number of the CFU, its value decreases with one logarithmic unit to the concentration of 156 μgL/mL (Figure 3); at concentrations greater than 156 μgL/mL, the CFU decreases with nine logarithmic units.

**Figure 4:**
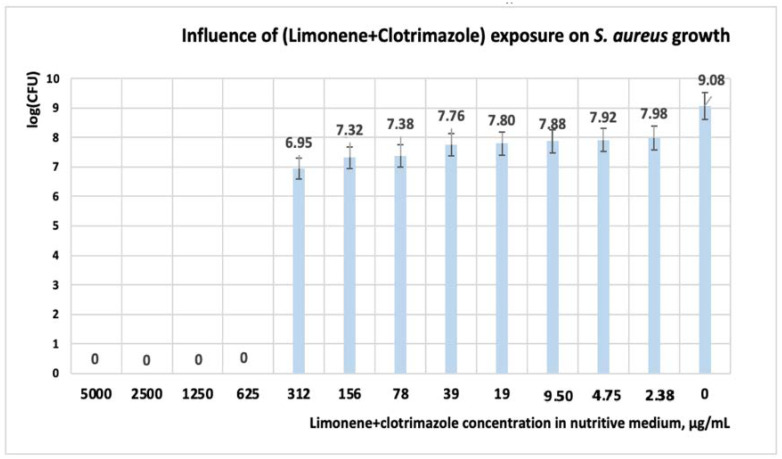
Influence of (limonene + CT) exposure on *S. aureus* growth, after 24 h. At a concentration of clotrimazole in the culture medium higher than 312 μg/mL, *S. aureus* is inhibited.

Other scientists obtained similar results on commercial CT formulation [22,43,44]. Kalhapure et al. have reported that after 18 h of exposure to CT, a MIC = 31.25 μgL/mL is obtained for both *S. aureus* and *S. aureus* MRSA [22]. In experiments performed by the Kirby Bauer method, El-Halim et al. reported the antibacterial activity of CT solutions with concentrations in the range of (1000 ÷ 5000) μg/mL [41]. Analyzing the influence of the mixture between limonene and CT, the interval of concentration at which *S. aureus* is susceptible, is situated in the range of (5000 ÷ 625) μg/mL (Table 4 and Table 5, Figure 4).

When concentrations of a mixture of (312 ÷ 78) μg/mL (Table 4) are used, OD ranges between 0.03 and 0.08 units, and we have considered that, at these concentrations, the microorganisms are intermediate (Table 4, Figure 4). If the concentration of the mixture is less than 78 μg/mL, the microorganism is resistant. Regarding the number of the CFU, its value decreases with one logarithmic unit to the concentration of 312 μg/mL (Figure 4). If the mixture concentrations are greater than 312 μg/mL, the CFU decreases with 7 log units, and the cell growth is inhibited. Interestingly, under the influence of the mixture between limonene and CT, the CFU is lower compared to experimental variants in which each reagent is used alone. The mixture concentrations of (156 ÷ 78) μg/mL are interesting because if the reagents are used individually, then the microorganism is resistant (Table 5); however, in the presence of both reagents, the microorganism has intermediate susceptibility (I).

#### 3.1.3. Studies on Microplates Performed on *C. albicans*

**Figure 5:**
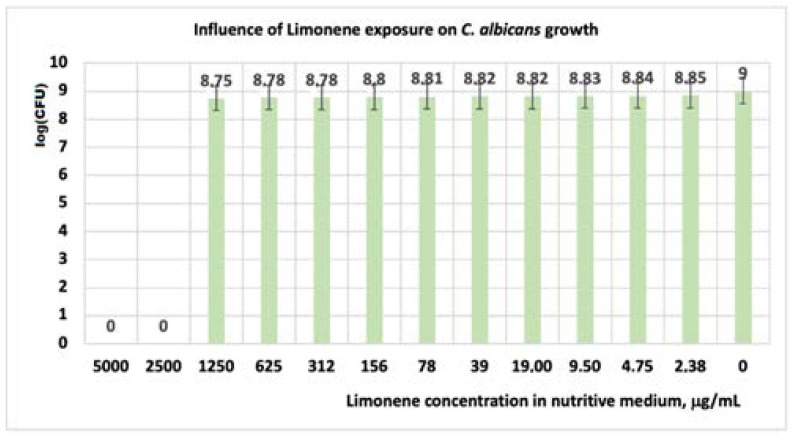
Influence of limonene exposure on *C. albicans* growth, after 48 h. At a concentration of limonene in the culture medium higher than 1250 μg/mL, *C. albicans* is inhibited.

**Figure 6:**
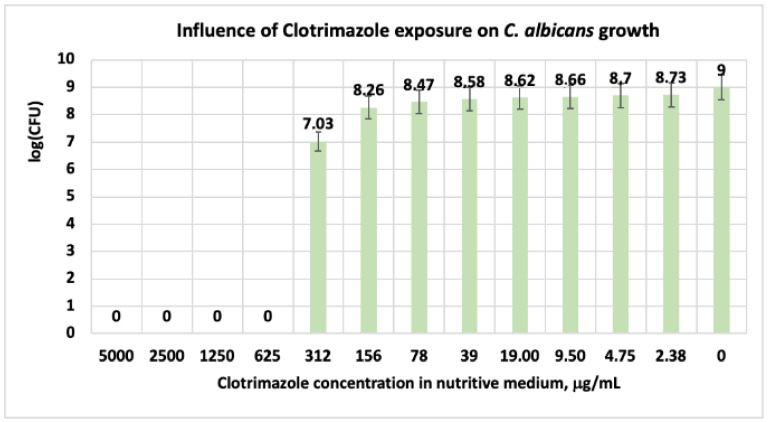
Influence of CT exposure on *C. albicans* growth, after 48 h. At a concentration of clotrimazole in the culture medium higher than 312 μg/mL, *C. albicans* is inhibited.

In the case of *C. albicans*, the obtained results revealed that, under the influence of limonene, the cell growth is inhibited at concentrations of 5000 ÷ 2500 μg/mL (Table 6 and Table 7, Figure 5 and Figure 6). For these concentrations, in our experimental model, the microorganism is susceptible (Table 7). At concentrations of limonene less than 2500 μg/mL, *C. albicans* is resistant. In our experimental model, the obtained values showed that the reduction of the CFU at limonene concentrations of (0 ÷ 1250) μg/mL is made slowly (Figure 5).

Similar results were obtained in studies performed by Thakre et al. with limonene in the concentrations range of 0.6–20 mM, on *C. albicans* ATTC 10231 [45]. Authors reported that at a concentration of 20 mM limonene in a culture medium, the microorganism growth, the biofilm development, and the biofilm maturation can be reduced substantially up to 90%. Nidhi and collab. have reported that, when using essential oils with 28% limonene obtained from *Citrus aurantium* (bitter orange), on two types of *C. albicans*, they obtained a MIC value of 0,15% and 0.31%, respectively [11]. Regarding the clotrimazole effect on *C. albicans*, in our experimental model, at concentrations in the range of (5000 ÷ 625) μg/mL, the microorganism is susceptible. At a CT level of 312 μg/mL, the microorganism has intermediate susceptibility (I) (Table 6 and Table 7). At this level of CT, in the system, the number of the CFU decreases with two logarithmic units (Figure 6, Table 7).

**Figure 7:**
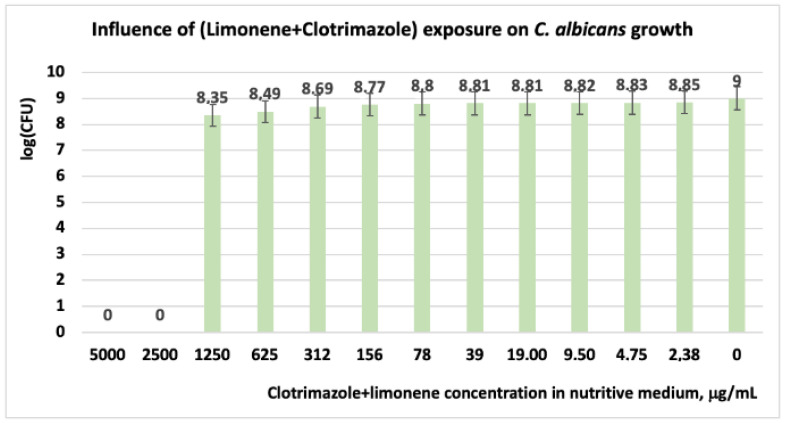
Influence of (CT + limonene) exposure on *C. albicans* growth, after 48 h. At a concentration of limonene + clotrimazole in the culture medium higher than 1250 μg/mL, *C. albicans* is inhibited.

For CT concentrations situated below 312 μg/mL, the *C. albicans* cells are resistant. Similarly, in experiments performed on *C. albicans*, other scientists have indicated MIC and MLC (minimum lethal concentration) values greater than 128 μg/mL [10,46]. Other researchers in studies performed on microorganisms implied in skin disease, with different antibiotic formulations from the market, revealed that the products with 1% CT are effective against infection with *C. albicans* [44,47]. In experiments performed with a mixture of (limonene + CT) on *C. albicans*, the results revealed that the microorganism is susceptible only at concentrations in the range of (5000 ÷ 2500) μg/mL (Table 4 and Table 5). At concentrations situated below these values, *C. albicans* is resistant. Regarding the number of the CFU in the system, these values are relatively close in the range of CT concentrations ranging between 0 and 1250 μg/mL (Figure 7).

### 3.2. Molecular Docking Studies

#### 3.2.1. Docking Studies Performed on *C. albicans*

**Figure 8:**
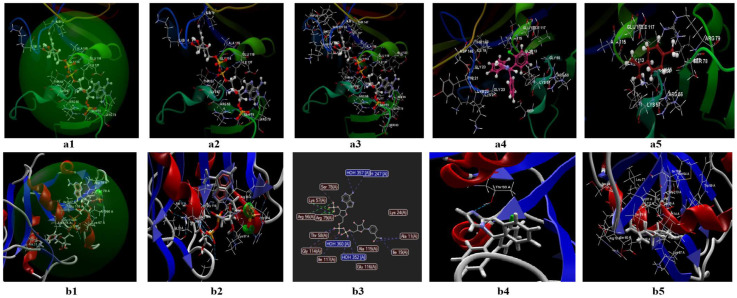
Molecular docking of the co-crystallized (NDP), limonene, and clotrimazole for *C. albicans* in the binding site 1AI9. (**a1**) Co-crystallized NDP (score: −79.35; RMSD 2.86) in the binding site (model generated by CLC); (**b1**) the co-crystallized NDP (score: −155.24; RMSD 0.00) in the binding site (model generated with MOLEGRO); (**a2**) hydrogen bonds of the co-crystallized NDP in the binding site, generated by CLC; (**b2**) hydrogen bonds, electrostatic interactions, steric interactions between the co-crystallized NDP and amino acids in 3D, generated by MOLEGRO; (**a3**) the interaction of co-crystallized NDP with amino acids’ residues in binding site, generated by CLC; (**b3**) hydrogen bonds (blue), electrostatic interactions (green), steric interactions (red) between the co-crystallized NDP and amino acids in 2D, generated by MOLEGRO; (**a4**) the interaction of clotrimazole with the amino acid residues from the binding site (model generated by CLC); (**b4**) the interactions of clotrimazole and the hydrogen bonds (blue line) with THR 58 amino acid in the binding site (model generated by MOLEGRO); (**a5**) the interaction of limonene with the amino acid residues from the binding site in a model generated with CLC; (**b5**) the interaction of limonene with the amino acid residues from the binding the site (model generated by MOLEGRO).

The two programs validate a protein fragment of *C. albicans* for which the reference ligand is possible to be fitted, in the active binding site of 1AI9 (Figure 8(a1,a2,b1,b2)). In both prediction models, the docking score of the two ligands (clotrimazole; limonene) is lower (in absolute value) than the docking score of the co-crystallized (Table 8). In the CLC model, the co-crystallized (NDP) creates a total of 16 hydrogen bonds as well as other interactions with amino acids of the binding site (Figure 8(a2,a3)), from which there are three bonds with ARG79, three bonds with ALA115, two bonds with THR 58, two hydrogen bonds with GLU 116, and two bonds with LYS 57. In the case of the studied ligands (clotrimazole, and limonene), the two prediction models do not detect the presence of hydrogen bonds (Table S1 in Supplementary), but they predict interactions with amino acids from the binding site (Figure 8(a4,a5)). The docking score of the studied compounds generated by the CLC prediction model in the absolute value decreases in the following order: limonene (docking score −41.94; RMSD: 0.01 Å) < clotrimazole (docking score: −57.87; RMSD: 0.10 Å) < co-crystallized NDP (docking score: −79.35; RMSD: 2.86 Å) (Table 8).

In the Molegro model (Figure 8(b1–b4)), the co-crystallized generates 20 hydrogen bonds (Figure 8(b2)), of which 4 are with ALA, 4 bonds with H_2_O, 3 bonds with ARG, 2 bonds with THR, and 2 bonds with GLU (data presented in Table S1 in Supplementary).

In the case of clotrimazole, the model identifies a single hydrogen bond with THR (Figure 8(b3)) and interactions between about 16 amino acids (Figure 8(b3–b5)) (details presented in the table from the Annex). For limonene, the predictive model does not identify hydrogen bonds but indicates the existence of interactions between 12 amino acids (Figure 8(b5)). The docking score obtained in this model, in absolute value, decreases in the following order: limonene (docking score −41.94; RMSD: 0.00 Å) < clotrimazole (docking score −77.75; RMSD: 0.00 Å) < co-crystallized NDP (docking score −155.24; RMSD: 0.00 Å).

Negative docking scores obtained in both models show the following aspects:

1. The two ligands (limonene; clotrimazole) are fitting in the binding site predicted by the two models;
2. Biological activities of the two ligands (clotrimazole; limonene) are similar but smaller than co-crystallized NDP;
3. According to Lipinski’s rule of five [35], the values generated by the CLC model for the three ligands studied (clotrimazole, limonene, and the co-crystallized) are more favorable for delivery into the cell for limonene and clotrimazole (molecular mass less than 500) in comparison with co-crystallized (molecular mass 739.37 Da). In addition, the value obtained for the partition coefficient in octanol (logP) is more favorable for clotrimazole (logP = 3.36) and the co-crystallized (log P = −4.72) (Table 9). Analyzing the value of the partition coefficient (logP) in the two ligands (Table 9), it can be appreciated that in the case of a mixture at low concentrations between the two compounds (i.e., clotrimazole; limonene), the presence of clotrimazole is determinant in transdermal delivery, due to the absence of the hydrogen bond [48,49,50,51], and more likely will be docked first.

#### 3.2.2. Docking Studies Performed on *S. aureus*

**Figure 9:**
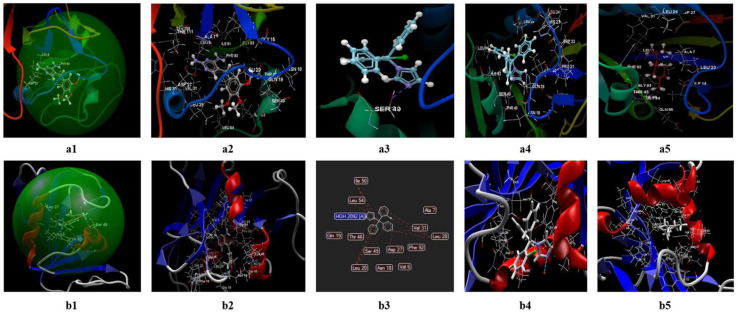
Molecular docking of co-crystallized (TOP), limonene, and clotrimazole for *S. aureus* in binding site 2W9H. (**a1**) Docking of the co-crystallized TOP (score: −53.12; RMSD 0.35) in the binding site of 2W9H (model generated by CLC); (**b1**) docking of the co-crystallized TOP (score: −100.32; RMSD 0.00) in the binding site of 2W9H (model generated by MOLEGRO); (**a2**) interactions between the co-crystallized TOP and the amino acid residues in the binding site 2W9H (model generated by CLC). The blue lines represent the hydrogen bonds; (**b2**) interactions between the co-crystallized TOP and the amino acid residues in the binding site of 2W9H (model generated with MOLEGRO). The blue lines represent the hydrogen bonds; (**a3**) hydrogen bonds (blue dotted lines) between clotrimazole and SER 49 amino acid in the binding site 2W9H, generated by CLC; (**b3**) hydrogen bonds (blue) and steric interactions (red) between clotrimazole and amino acids in the binding site 2W9H in the 2D format, generated by MOLEGRO; (**a4**) the interactions between the clotrimazole and the amino acid residues in the binding site 2W9H, generated by CLC; (**b4**) hydrogen bonds and steric interactions, between clotrimazole and the amino acids, in the binding site 2W9H, generated by MOLEGRO; (**a5**) interactions of limonene with the amino acid residues in the binding site of 2W9H, generated by CLC; (**b5**) interactions of limonene with the amino acid residues in the binding site 2W9H, generated by MOLEGRO.

In the case of studies performed in silico on *S. aureus*, the programs validate the binding site named 2W9H, in which the ligands’ co-crystallized TOP, clotrimazole, or limonene are possible to be fitted (Figure 9(a1,b1)). In the CLC model (Table S2 in Supplementary, Figure 9(a1–a5)), the co-crystallized TOP creates a total of six hydrogen bonds, from which two bonds are with ASP27, two bonds with SER49, one bond with PHE 92, one bond with LEU5, and two bonds with LYS 57 (Table S2 in Supplementary, Figure 9(a2)). Here are the possible the interactions between the co-crystallized and the rest of the 18 amino acids from the binding site. Regarding clotrimazole and limonene, in the CLC model, clotrimazole generates one hydrogen bond with SER 49 (Figure 9(a3)) and 13 interactions with amino acids from the binding site (Figure 9(a4)); limonene can generate interactions with 15 amino acids from the binding site (Figure 9(a5)). The docking score of the studied compounds decreases in the following order: limonene (docking score −39,63; RMSD: 0.02 Å) > clotrimazole (docking score: −47.56.87; RMSD: 0.13 Å) > co-crystallized TOP (docking score: −53.12; RMSD: 0.35 Å). In the CLC model, clotrimazole can interact with 13 groups of amino acids, and limonene with 15 groups. In the Molegro model (Figure 9(b1–b5)), the co-crystallized generates interactions with the rest of the 18 amino acids of the binding site (Figure 9(b2)) and 8 hydrogen bonds, from which 4 bonds are with H_2_O, and 4 bonds with 4 amino acids. In the case of clotrimazole, the model identifies a single hydrogen bond with H_2_O 2092, and interactions between about 13 amino acids (tables from the Annex; Figure 9(b3,b4)). For limonene, the predictive model does not identify hydrogen bonds, but indicates the possibility of interactions between 11 amino acids (Figure 9(b5)). The docking score obtained in this model decreases in the following order: limonene (docking score −50.58; RMSD: 0.00 Å) > clotrimazole (docking score −59.82; RMSD: 0.00 Å) > co-crystallized TOP (docking score −100,53; RMSD: 0.00 Å). Negative docking scores obtained in both models suggest the following:

- Both ligands (limonene, clotrimazole) can be fitted in the binding site predicted by the two models;
- The partition coefficient is comparable with the co-crystallized (logP= 3.46) in the case of clotrimazole (logP = 3.36) (table from the Annex), but greater than TOP in the case of limonene (logP = 5.41);
- It is not clear if limonene and clotrimazole can act synergically.

#### 3.2.3. Docking Studies Performed on *E. coli*

**Figure 10:**
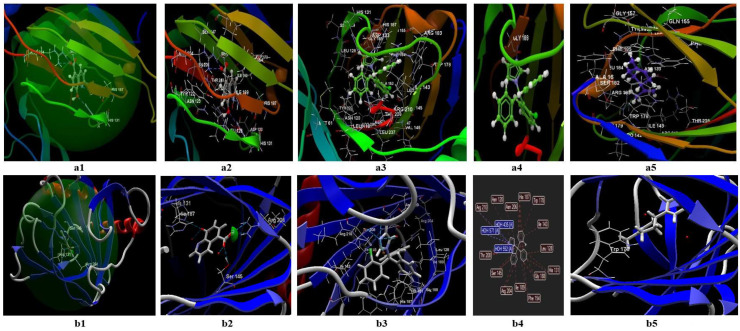
Molecular docking of the co-crystallized 8XQ, limonene, and clotrimazole for *E. coli* in the binding site 4JHT. (**a1**) Docking of the co-crystallized 8XQ in the binding site 4JHT generated by CLC); (**b1**) docking of the co-crystallized in the binding site 4JHT, generated by MOLEGRO); (**a2**) docking of the co-crystallized 8XQ, hydrogen bonds, and interactions with amino acid residues from the binding site 4JHT, generated by CLC; (**b2**) hydrogen bonds, electrostatic interactions, and steric interactions between the co-crystallized 8XQ and the amino acids from the binding site 4JHT, in 3D model generated by MOLEGRO; (**a3**) interactions between clotrimazole and the amino acid residues from the binding site 84JHT, generated by CLC; (**b3**) hydrogen bonds and steric interactions between clotrimazole and the amino acids from the binding site 4JHT, generated with MOLEGRO; (**a4**) the hydrogen bonds (blue dotted lines) between clotrimazole and SER 49, in the binding site 4JHT, generated by CLC; (**b4**) the hydrogen bonds (blue) and steric interactions (red) between clotrimazole and the amino acids from the binding site 4JHT, 2D model generated by MOLEGRO; (**a5**) the interactions of limonene with the amino acid residues from the binding site 4JHT, generated by CLC; (**b5**) the interactions of limonene with the amino acid residues from the binding site 4JHT, generated by MOLEGRO.

In the case of *E. coli*, the two programs validate the binding site named 4JHT (Figure 10(a1,b1)).

In the CLC Drug Discovery Workbench model, the co-crystallized 8XQ creates a total of five hydrogen bonds from which three bonds are with ASP, one bond with TYR, one bond with TRP, and fifteen possible interactions with amino acids from the binding site (Table S3 in Supplementary, Figure 10(a2)). Regarding clotrimazole and limonene, in the CLC model (Figure 10(a1–a5)) clotrimazole generates 1 hydrogen bond with GLY188 and 27 other possible interactions with amino acids from the binding site (Figure 10(a3,a4)). Limonene does not generate hydrogen bonds but can interact with 18 groups of amino acids (Figure 10(a5)). The docking score of the studied compounds decreases in the following order: clotrimazole (docking score −10,06; RMSD: 0.01 Å) > 8XQ (docking score: −37,48; RMSD: 0.03 Å) > limonene (docking score: −45.14; RMSD: 0.12 Å) (Table S3 in Supplementary).

In the Molegro model (Table S3 in Supplementary, Figure 10(b1–b5)), the co-crystallized generates 18 interactions with the rest of the amino acids from the binding site and 6 amino acids (Figure 10(b1,b2)). In the case of clotrimazole, the model identifies 6 hydrogen bonds and 24 interactions between amino acids (Table S3 in Supplementary, Figure 10(b3,b4)). For limonene, the predictive model does not identify hydrogen bonds but indicates the possibility of interactions with 17 amino acids (Figure 10(b5)). 

The docking score obtained in this model decreases in the following order: clotrimazole (docking score −64.85; RMSD: 0.00 Å) > limonene (docking score −65.79; RMSD: 0.00 Å) > co-crystalized 8XQ (docking score −79.94; RMSD: 0.00 Å).

Negative docking scores obtained in both models suggest the following:

- The ligands can be fitted in the binding site predicted by the two models;
- The partition coefficients of the co-crystallized (logP = 2.44) are smaller than clotrimazole (logP = 3.36) (Table 9) and limonene (logP = 5.41).

Due to the similar docking score obtained in both models, the great number of interactions with amino acids from the binding site, and the low number of Lipinski’s violations for clotrimazole, this ligand can be docked first in the binding site. The synergy between the two ligands (limonene; clotrimazole) in the case of *E. coli* is less probable.

*a.*  *Validating the Antimicrobial Activities by Tests Performed “In Vitro”*

**Figure 11:**
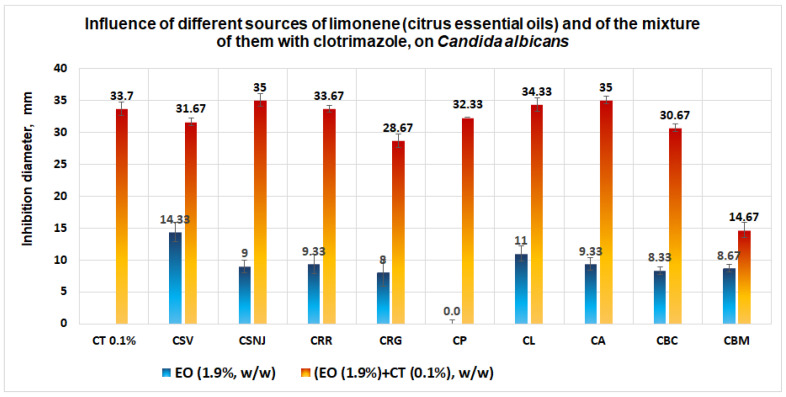
Influence of different sources of limonene (citrus essential oil, 1.9%, w/w) and of the mixture of them with clotrimazole (0.1%, w/w), on *C. albicans*. No synergism between clotrimazole and the different sources of limonene was observed.

In the case *of C. albicans*, the best results are obtained for the mixture between the essential oil from *Citrus sinensis* (NJoy) and clotrimazole, and, respectively, in the case of the mixture between the essential oil from *Citrus aurantium* and clotrimazole (diameter of inhibition obtained is 35 mm), followed by the mixture between *Citrus limone* and clotrimazole (diameter of inhibition obtained is 34.33 mm) (Figure 11).

**Figure 12:**
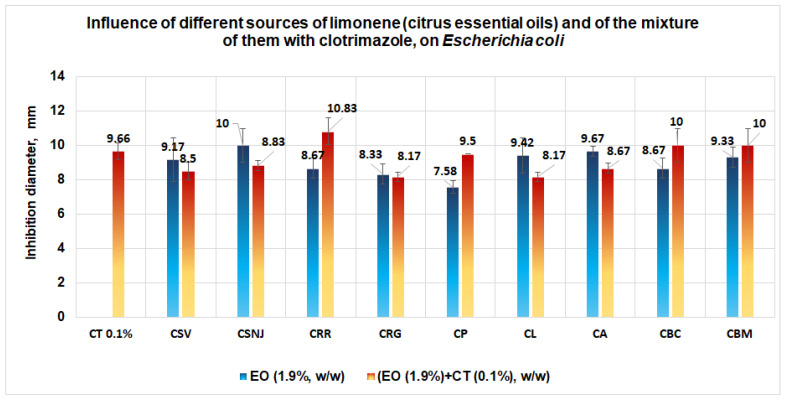
Influence of different sources of limonene (citrus essential oil, 1.9%, w/w) and of the mixture of them with clotrimazole (0.1%, w/w), on *E.coli.* No synergism between clotrimazole and the different sources of limonene was observed.

Regarding *E. coli*, an easily increased antimicrobial activity appears in the case of the mixture between the essential oil from *Citrus reticulata* (red mandarin) and clotrimazole (diameter of inhibition obtained is 10.83 mm), followed by the mixture between clotrimazole and the essential oil from *Citrus bergamia* (supplied by Life and Mayam) when an inhibition diameter of 10 mm is obtained (Figure 12).

**Figure 13:**
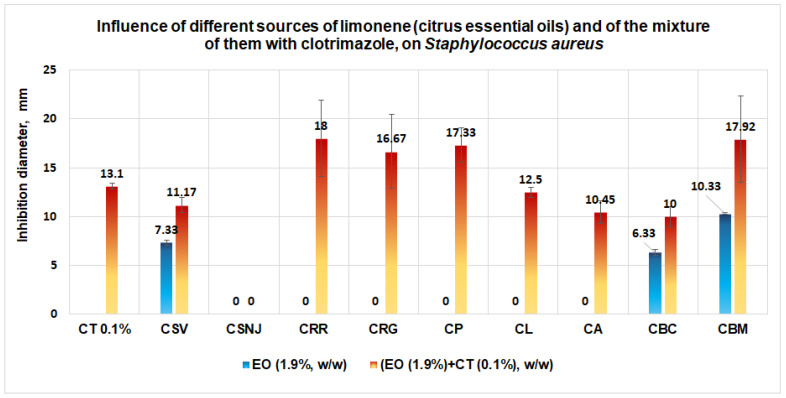
Influence of different sources of limonene (citrus essential oil, 1.9%, w/w) and of the mixture of them with clotrimazole (0.1%, w/w) on *S. aureus.* The synergistic effect is evident in the case of the mixtures between clotrimazole and the essential oils of mandarin (red mandarin or green mandarin), and, respectively, between clotrimazole and the essential oil of grapefruit.

A good performance is obtained in the case of *S. aureus* when the antimicrobial activity increases slightly in the case of the mixture between clotrimazole and the essential oil from *Citrus reticulata* (red mandarin) when an inhibition diameter of 18 mm is obtained (Figure 13). In second place is the mixture between clotrimazole and the essential oil from *Citrus bergamia* (Mayam) (diameter of inhibition is 17.92 mm); in third place is the mixture between clotrimazole and the essential oil from *Citrus paradisi* (17.33 mm inhibition diameter), and, finally, the mixture between clotrimazole and the essential oil from *Citrus reticulata* (green mandarin) with an inhibition diameter of 16.67 mm.

**Figure 14:**
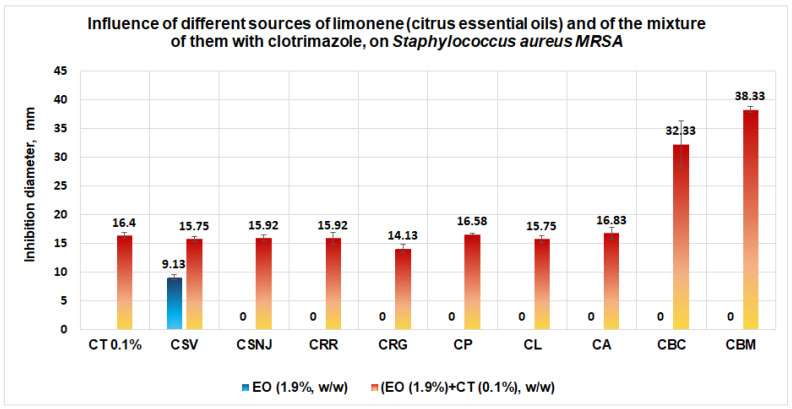
Influence of different sources of limonene (citrus essential oil, 1.9%, w/w) and of the mixture of them with clotrimazole (0.1%, w/w), on *S. aureus MRSA.* The synergistic effect is evident in the case of the mixtures between clotrimazole and the essential oils of bergamot (two sources of this essential oil).

Good results are obtained in the case of *S. aureus* MRSA (Figure 14), for which generally the citrus essential oils in the concentration of 1.9% (w/w) do not have any antimicrobial effect, except the essential oil from *Citrus sinensis* (orange of Valencia), for which an inhibition diameter of 9.13 mm is obtained. In comparison with the solution of clotrimazole 0.1% that gives an inhibition diameter of 16.4 mm, the mixture between clotrimazole and the essential oils of Citrus bergamia, Citrus aurantium, Citrus paradisi, and Citrus limone give the inhibition diameters greater than each essential oil or CT. The best antimicrobial activities against *S. aureus* MRSA are obtained for a mixture of clotrimazole and the two essential oils of *Citrus bergamia*, (supplied by Life and Mayam), when the average inhibition diameters obtained are situated between 32.33 mm and 38.33 mm.

**Figure 15:**
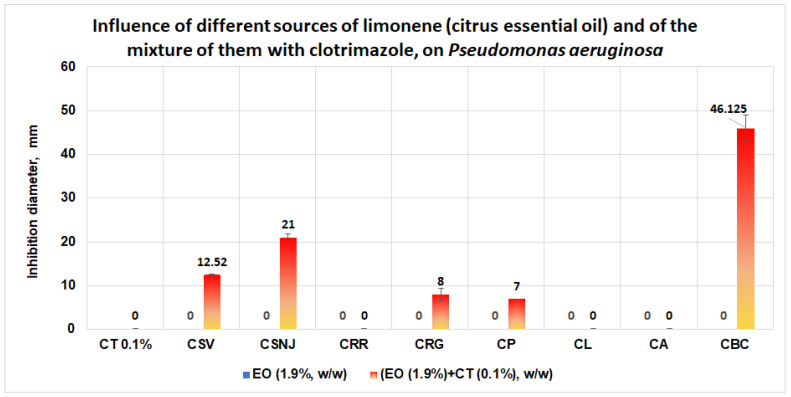
Influence of different sources of limonene (citrus essential oil, 1.9%, w/w) and of the mixture of them with clotrimazole (0.1%, w/w) on *P. aeruginosa.* The synergistic effect is evident in the case of the mixtures between clotrimazole and essential oils of *Citrus bergamia* and *Citrus sinensis*.

In the case of *P. aeruginosa*, the results obtained are more spectacular. The clotrimazole and the citrus essential oil used alone do not have any antimicrobial effect on this microorganism. However, in the case of the mixture of clotrimazole and the essential oil from *Citrus bergamia* (Mayam), an average inhibition diameter of 46.125 mm is obtained (Figure 15). In the case of *P. aeruginosa*, good performances are obtained in the case of the mixtures between clotrimazole and the essential oils of *Citrus sinensis* (Life, nJoy), which give the inhibition diameters of 12.52 mm and, respectively, 21 mm. Local antimicrobial activities are registered in the case of the mixture between clotrimazole and the essential oil from *Citrus reticulata* (green mandarin) or *Citrus paradisi*, when the inhibition diameters obtained are 8 mm and 7 mm, respectively.

## 4. Discussion

If we analyze the results obtained from the studies performed on microplates in terms of interaction types between clotrimazole and each target (microorganism), taking into account the values obtained after calculating the fractional inhibitory concentration index (FICI), we obtain an indifference effect in the case of *C. albicans* and, respectively, a synergism effect in the case of *S. aureus* (Table 10). In the case of *E. coli*, the effect can be due to the processes of addition and/or synergy, due to the close value obtained for FICI (0.4926) [52]. Regarding docking studies, in the case of *C. albicans*, both models give a negative score of docking for limonene and CT; however, these values are less than the co-crystallized (NDP) in both models. The number of hydrogen bonds formed between studied molecules in both models is less than 1. Both models identified more interactions between analyzed molecules and the rest of the amino acids from the binding site (1AI9). Due to the near value of the docking score for clotrimazole and the co-crystallized, we can conclude that in the case of a mixture between limonene and clotrimazole, the antibiotic reagent will be docked preferentially.

This supposition is confirmed by studies performed on Petri plates, where in the case of *C. albicans*, all inhibition diameters obtained in the case of a mixture between different sources of limonene and clotrimazole are closer as a mathematical value to clotrimazole, without a synergistic effect.

In the case of *E. coli*, both docking models give negative docking scores for limonene and clotrimazole, which means that both molecules can interact with the binding site (8XQ). The CLC model indicates the formation of one hydrogen bond between clotrimazole and the binding site, whereas the MOLEGRO model indicates the formation of six hydrogen bonds between the antibiotic reagent and the binding site. Regarding the interactions with the rest of the amino acids from the binding site, both models indicate a great number of interactions between clotrimazole and the rest of the amino acids from the binding site (24 possible interactions identified by Molegro models, and 27 interactions identified by the CLC model).

If we take into account only this information, it suggests that the antibiotic reagent will be docked first. A careful analysis of the values of the docking scores shows that both models give a value close to the co-crystallized for limonene; the CLC model gives a docking score of −45.14 (the co-crystallized docking score in this model is −34.48), whereas the Molegro model gives a docking score of −65.79 (the co-crystallized docking score in the Molegro model is −79.94). Taking into account only the values of the docking score, the molecule of limonene will be docked first. Applying Lipinski’s rule of five in this case indicates that limonene is docked first because its molecular mass is less than that of clotrimazole and of the co-crystallized.

In this case, we suppose that in the case of *E. coli*, a competition for the binding site between limonene and clotrimazole exists, without a clear conclusion. The studies performed on Petri plates with clotrimazole (0.1%) and different sources which contain limonene reveal that the inhibition diameters are closer to clotrimazole, without a synergistic effect. The value of inhibition diameter obtained in the case of both essential oils from *Citrus bergamia* (inhibition diameter of 10 mm) is probably due to the linalool content (CBM: 20.83%; CBC 23.31%) [53,54,55].

In conclusion, in the case of *E. coli*, the effect of the clotrimazole and limonene is most probably obtained by “Addition” and not by “Synergy”.

In the case of *S. aureus*, studies performed in microplates indicate synergism in the case of a mixture between clotrimazole and limonene (FICI = 0.0159). Docking studies performed on the two molecules with both programs (CLC and Molegro) reveal that docking scores are favorable for the two molecules. Studies performed on this microorganism with a mixture of clotrimazole (0.1%) and various sources of citrus essential oils (1.9%) reveal a clear synergistic effect in the case of different sources of limonene, such as essential oils from *Citrus reticulata* (red), *Citrus reticulata* (green), and *Citrus paradisi*, probably because these essential oils contain limonene and or/linalool (Citrus paradisi essential oil) [56,57]. In agreement with the results obtained by Guimarães and collab., beta-pinene and gamma-terpinene do not exhibit antimicrobial activity on *S. aureus* [56].

In the validation studies, noticeable results are obtained in the case of *S. aureus* MRSA, for which clearer synergistic effects are obtained in the case of a mixture between clotrimazole and the following citrus essential oils: *Citrus bergamia* (Life and Mayam supplier). *Citrus aurantium*, *Citrus limone*, and *Citrus paradisi.* Spectacular results are obtained with the essential oils of *Citrus bergamia* (two sources), which give the inhibition diameters of 32.33 mm and 38.33 mm.

These spectacular results are due to favorable reports between linalool and linalyl acetate (bergamole) from the bioproducts which are responsible for synergism in antimicrobial activity for *S. aureus* MRSA [58,59,60]. A synergistic effect from linalool and antibiotic reagent gentamicin is found in the case of *S aureus*, *S. aureus* MRSA. The synergistic effect is observed also in the study performed on *P. aeruginosa* for which a clear synergism is obtained in the case of the mixture between clotrimazole (0.1%) and essential oils from *Citrus sinensis* (supplier by Life or NJoy), and *Citrus bergamia*, for the last one with an exceptional result (inhibition diameter of 41.25 mm).

In the case of the mixture between the essential oil of *Citrus bergamia* and clotrimazole, the results are due to the presence of linalool and linalyl acetate, which destroy cell membrane, change the nucleotides’ integrity, and interfere with cellular respiration [61]. In the case of *P. aeruginosa*, an additive interaction was reported by Adaszyńska-Skwirzyńska and collab. in the case of a mixture between linalool and gentamicin [61].

In fact, these studies represent just a proof of concept that the mixture of different sources of limonene and clotrimazole can enhance antimicrobial activity. In neither case, will the mixture of essential oils and clotrimazole in propylene glycol represent the end pharmaceutical formulation. Studies are still in progress regarding the formulations which contain limonene and clotrimazole. In one of these studies, we developed the solid formulations with clotrimazole [62] as a sponge, with collagen, chitosan, and clotrimazole. Here, the studies performed in vitro on normal cell lines confirm that the matrix used to obtain end products diminishes the toxicity of the formulations with clotrimazole.

## 5. Conclusions

Studies performed in vitro on microplates in an experimental model made with limonene, clotrimazole, and a mixture of them on three types of microorganisms involved in skin diseases revealed the following: in the case of *E.coli*, the mixture between CT and limonene decreases the range of concentration at which this microorganism is susceptible, at levels in the range of (19 ÷ 9.5) μg/mL; in the case of *S. aureus*, results showed that it is susceptible to increased exposure at levels of concentrations in the range of 156 ÷ 78 μg/mL; the mixture between CT and limonene has no influence on *C. albicans’* growth.

Docking studies performed using CLC Drug Discovery Workbench and MOLEGRO Virtual Docker showed the following:

- Regarding *C. albicans*, data obtained suggest that, in the case of a mixture between limonene and clotrimazole, in small quantities, clotrimazole is docked first;
- Data for *S. aureus* show a similar docking score using both models and suggest that the limonene will be docked first in the binding site. Synergism between the two ligands (limonene; clotrimazole) in the case of *S. aureus* is more probable;
- In the case of *E. coli*, taking into account the similar docking data obtained in both models, the great numbers of interactions with amino acids from the binding site, and zero Lipinski’s violations for clotrimazole, it can be assumed that this ligand will be docked first in the binding site. The synergism between the two ligands (i.e., limonene; clotrimazole) in the case of *E. coli* is less probable. For *E. coli*, the effect of the mixture between clotrimazole and limonene is probably made by addition;
- Results obtained in validation studies indicate a clear synergism between the mixture of clotrimazole and different sources of limonene (citrus essential oils) against *S. aureus*, *S aureus* MRSA, and *P. aeruginosa*, with the best results for the essential oil of *Citrus bergamia* for *S aureus* MRSA and *P. aeruginosa.* The studies performed are important because they:
  - Indicate the synergism between clotrimazole and limonene against *S. aureus*, in the case of sources with limonene with the content of limonene and/or linalool;
  - Show what the best natural resources for limonene are, in order to obtain a synergistic effect;
  - Reveal the importance of lab studies (performed “in vitro”), which must be in agreement with the results obtained “in silico”.

The future developments of this study consist of:

1. Obtaining pharmaceutical products with clotrimazole, limonene, and different sources of limonene that exhibit the best antimicrobial activity and low toxicity;
2. Testing these pharmaceutical products on pathogenic microorganisms involved in skin injury.

## Figures and Tables

**Table 1:** Natural source of limonene used to validate antimicrobial properties.

No Crt	Source of Limonene	*Code*	Major Compounds (w/w)	Minor Compounds w/w
1	Essential oils from *Citrus sinensis* (Raw Valencia orange). Supplier: Life	CSV	Limonene 96.94%	-
2	Essential oils from *Citrus sinensis.* Supplier: nJoy Nature	CSNJ	Limonene: 96%	β-Pinene: 2.07% Linalool: 1.04
3	Essential oil from *Citrus reticulata* (red mandarin). Supplier: Life	CRR	Limonene: 71.44% γ-Terpinene: 21.64%	β-Phellandrene: 1.16% α-Pinene: 1.63% o-Cymol: 1.86%
4	Essential oil from *Citrus reticulata* (green mandarin). Supplier: Life	CRG	Limonene: 67.56% γ-Terpinene: 20.8%	β-Pinene: 1.91% α-Pinene: 1.65% o-Cymene: 4.12%
5	Essential oils from *Citrus limone*. Supplier: Arom Sciences	CL	Limonene: 59.07% β-Pinene = 15.14% γ-Terpinene: 14.2%	β-Phellandrene: 3.04% α-Pinene: 2.79%
6	Essential oil from *Citrus paradisi*. Supplier: Adams Vision	CP	Limonene: 64.61% Bergamole: 14.12% Linalool: 9.45%	Linalool: 9.45% α-Pinene: 1.92%
7	Essential oils from *Citrus aurantium*. Supplier: Arom Sciences	CA	Limonene: 58.76% γ-Terpinene: 14.56% β-Pinene = 14.12%	β-Phellandrene: 2.84% α-Citral: 2.98%
8	Essential oil from *Citrus bergamia*. Supplier: Life	CBC	Limonene: 30.41% Bergamole: 28.92% Linalool: 23.31%	γ-Terpinene: 7.28% β-Pinene: 4.63
9	Essential oil from *Citrus bergamia*. Supplier: Mayam	CBM	Limonene: 25.01% Bergamole: 39.43% β-Linalool: 20.83%	γ-Terpinene: 4.56% β-Pinene: 4.26%
Major compounds: c ≥ 10%; minor compounds: 1 ≤ c < 10 Beragamole = linalyl acetate				

**Table 2:** Optical densities obtained after 24 h of exposure of *E. coli* at different treatments.

Microorganism: *E. coli*	Concentration, (μg·mL^−1^)												
	5000	2500	1250	625	312	156	78	39	19	9.5	4.75	2.37	0
Limonene	0	0	0	0	0	0	0	0	0	0.05	0.01	0.01	0.43
CT	0	0	0	0	0	0	0	0	0	0.08	0.06	0.20	0.43
Limonene + CT	0	0	0	0	0	0	0	0	0	0	0	0.117	0.43

**Table 3:** Susceptibility of *E. coli* at different treatments.

Microorganism: *E. coli*	Concentration, (μg·mL^−1^)											
	5000	2500	1250	625	312	156	78	39	19	9.5	4.75	2.37
Limonene	S	S	S	S	S	S	S	S	I	I	I	R
CT	S	S	S	S	S	S	S	S	I	I	R	R
Limonene + CT	S	S	S	S	S	S	S	S	S	S	R	R

Susceptible (S): no growth (OD = 0); susceptible, to increased exposure, i.e., intermediate susceptibility (I): OD < 0.1; resistant: OD ≥ 0.1.

**Table 4:** Optical densities obtained after 24 h of exposure of *S. aureus* at different treatments.

Microorganism: *S. aureus*	Concentration, (μg·mL^−1^)												
	5000	2500	1250	625	312	156	78	39	19	9.5	4.75	2.37	0
Limonene	0	0.007	0.663	0.711	0.78	1.154	1.21	1.29	1.35	1.52	1.88	2.02	3
CT	0	0	0	0	0	0.379	0.40	0.49	0.53	0.58	0.62	0.71	3
Limonene + CT	0	0	0	0	0.03	0.07	0.08	0.19	0.21	0.25	0.28	0.32	3

**Table 5:** Susceptibility of *S. aureus* at different treatments.

Microorganism: *S. aureus*	Concentration, (μg·mL^−1^)											
	5000	2500	1250	625	312	156	78	39	19	9.5	4.75	2.37
Limonene	S	I	R	R	R	R	R	R	R	R	R	R
C	S	S	S	S	S	R	R	R	R	R	R	R
Limonene + CT	S	S	S	S	I	I	I	R	R	R	R	R

Susceptible (S): no growth (OD = 0); susceptible, to increased exposure, i.e., intermediate susceptibility (I): OD < 0.1; resistant: OD ≥ 0.1.

**Table 6:** Optical densities obtained after 48 h by exposure of *C. albicans* at different treatments.

Microorganism: *C. albicans*	Concentration, (μg·mL^−1^)												
	5000	2500	1250	625	312	156	78	39	19	9.5	4.75	2.37	0
Limonene	0	0	8.75	8.78	8.78	8.80	8.81	8.82	8.82	8.83	8.84	8.85	9
CT	0	0	0	0	7.03	8.26	8.47	8.58	8.62	8.66	8.70	8.73	9
Limonene + CT	0	0	8.35	8.49	8.69	8.77	8.80	8.81	8.81	8.82	8.83	8.85	9

**Table 7:** Susceptibility of *C. albicans* at different treatments.

Microorganism: *C. albicans*	Concentration, (μg·mL^−1^)											
	5000	2500	1250	625	312	156	78	39	19	9.5	4.75	2.37
Limonene	S	S	R	R	R	R	R	R	R	R	R	R
CT	S	S	S	S	I	R	R	R	R	R	R	R
Limonene + CT	S	S	R	R	R	R	R	R	R	R	R	R

Susceptible (S): no growth (OD = 0); susceptible, to increased exposure, i.e., intermediate susceptibility (I): OD < 0.1; resistant: OD ≥ 0.1.

**Table 8:** The docking score and root mean square deviation between the docked ligands on the specific binding sites from the three microorganisms.

Ligand	Score	RMSD, Å	Ligand	Score	RMSD, Å
The docking score and root mean square deviation between the docked ligands on the binding site 1AI9 of *C. albicans*.					
**Model generated by CLC**			**Model generated by MOLEGRO**		
Co-crystallized NDP	−79.35	2.86	Co-crystallized NDP	−155.24	0.00
Clotrimazole	−57.87	0.10	Clotrimazole	−77.75	0.00
Limonene	−41.94	0.01	Limonene	−73.71	0.00
The docking score and root mean square deviation between the docked ligands on the binding site 2W9H of *S. aureus*					
Co-crystallized TOP	−53.12	0.35	Co-crystallized TOP	−100.32	0.00
Clotrimazole	−47.56	0.13	Clotrimazole	−59.82	0.00
Limonene	−39.63	0.02	Limonene	−50.58	0.00
The docking score and root mean square deviation between the docked ligands on the binding site 4JHT of *E. coli*					
Co-crystallized 8XQ	−37.48	0.03	Co-crystallized 8XQ	−79.94	0.00
Clotrimazole	−10.06	0.01	Clotrimazole	−64.85	0.00
Limonene	−45.14	0.12	Limonene	−65.79	0.00

**Table 9:** Predicted properties of ligands generated by CLC Drug Discovery Workbench.

Compounds	Atoms	Weight [Daltons]	Flexible Bonds	Lipinski Violations	Hydrogen Donors	Hydrogen Acceptors	Log P
Co-crystallized NDP *	72	739.37	13	3	6	24	−4.72
Co-crystallized TOP **	39	290.32	5	0	4	7	3.46
Co-crystallized 8XQ ***	20	188.16	1	0	1	4	2.44
Clotrimazole	42	344.84	4	1	0	0	3.36
Limonene	26	136.23	1	0	0	2	5.41

* PDB ID: 1AI9; ** PDB ID: 2W9H; *** PDB ID: 4JHT.

**Table 10:** Evaluation of the effect of clotrimazole and limonene on *C. albicans*, *E. coli.*, and *S. aureus*.

Microorganism/Compound	MIC, μg/mL	Fractional Inhibitory Concentration, FIC	Fractional Inhibitory Concentration Index, FICI	Type of Interaction
***Candida albicans***				
Limonene	1250	0.2496		
Clotrimazole	312	1		
Limonene + Clotrimazole	312		1.2496	**Indifference**
***Escherichia coli***				
Limonene	9.5	0.2463		
Clotrimazole	9.5	0.2463		
Limonene + Clotrimazole	2.34		0.4926	**Synergy and/or Addition**
***Staphyloccocus aureus***				
Limonene	2500	0.0009		
Clotrimazole	156	0.015		
Limonene + Clotrimazole	2.34		0.0159	**Synergy**
FIC _limonene_ = MIC _mixture (limonene+clotrimazole)_/MIC _limonene_ FIC _clotrimazole_ = MIC _mixture (limonene+clotrimazole)_/MIC _clotrimazole_ FICI = FIC limonene + FIC clotrimazole FICI < 0.5: Synergy 0.5 ≤ FICI ≤ 1: Addition 1.1 < FICI ≤ 400: Indiference; FICI > 4: Antagonism				

## Data Availability

Not applicable.

## Supplementary Materials

The following supporting information can be downloaded at: https://www.mdpi.com/article/10.3390/antibiotics11121816/s1. Table S1: Intermolecular interactions between the ligands docked on 1AI9.; Table S2: Intermolecular interactions between the compounds docked on the binding site for *S. aureus;* Table S3: The list of intermolecular interactions between the compounds docked with 8XQ.

## Abbreviations

- Susceptible (S) (or sensitive)

The term is used in two senses, one microbiological and the other clinical.

Microbiological Susceptibility: Susceptible (sensitive) bacteria are those that belong to the most susceptible sub-populations and lack mechanisms of resistance.

Clinical Susceptibility: Susceptible (sensitive) bacteria are those that ideally have been shown to respond to a standardized therapeutic regimen when causing infection.

- Intermediate = a bacterium is classified with **intermediate** susceptibility if it belongs to the group of strains that lies between the clinically susceptible and the clinically resistant. Infections caused by such strains have variable (or indeterminate) responses to chemotherapy, but they may be eliminated if the antibiotic is concentrated at the site of the infection, or the dosage is increased.
- Resistance = this term is used in two senses, microbiological and clinical.

Microbiological resistance organisms are those that possess any resistance mechanism. The term may be qualified, as in “moderately or highly resistant” or “low-level, or high-level resistance”.

Clinical resistance occurs when the infection is highly unlikely to respond even to maximum doses of a given antibiotic.

- Co-crystallizate = native (natural) ligand.
- Score = the docking score used in the Drug Discovery Workbench is the PLANTSPLP score [63]. This score has a good balance between accuracy and evaluation time. The score mimics the potential energy change when the protein and ligand come together. This means that a very negative score corresponds to a strong binding; a less negative or even positive score corresponds to a weak or non-existent binding. (Score = S target-ligand + S ligand). The S target-ligand term is a sum of contributions from all heavy atom contacts between the ligand and the molecules included in the binding site setup. It scores the complementarity between the binding site and ligand by rewarding and punishing different types of heavy atom contacts (inter-atom distance below ~5.5 Å). Five different types of contacts are defined:

**Rewarded contacts:**

1. Hydrogen bond interactions
2. Lone–pair–metal ion interactions
3. Non-polar interactions

**Punished contacts:**

4. Non-polar–polar contacts
5. Repulsive contacts:

Hydrogen bond donor–donor contacts

Hydrogen bond donor–metal contacts

Hydrogen bond acceptor–acceptor contacts

These contact types are associated with a pairwise linear potential (PLP), which determines the distance dependence of the contribution to the score, -docking score–PLANTSPLP score [63].

- RMSD = root mean square deviation. The root mean square deviation (RMSD) is measured between the heavy atom positions of the ligand, in the same way as the docking between the co-crystallized and the binding site, with high accuracy (<2 Å RMSD).
- Group interaction: amino acid group within the binding site, potentially interacting with the ligand.
