# Supplementary material for: Studies Regarding the Antimicrobial Behavior of Clotrimazole and Limonene
Source: Antibiotics (Basel). 2022 Dec 14;11(12):1816. doi: 10.3390/antibiotics11121816 (PMC9774930; doi:10.3390/antibiotics11121816)
Supplement: Supplementary file 1 [file antibiotics-11-01816-s001.zip › antibiotics-2067641-supplementary.pdf]

Anexes

Table S1 Intermolecular interactions between the ligands docked on 1AI9.

Ligand	Score	RMSD*,Å	Group interaction	Hydrogen bond	Bond length, Å
**The model generated by CLC Drug Discovery Workbench**					
Co-crystallized (NDP)	-79.35	2.86	LEU 121, GLU 120, SER 95, TYR 118, GLU 116, ILE 117, SER 94, ARG 79, ILE 9, ALA 93, VAL 10, SER 78, LEU 77, ILE 112, GLY 113, MET 54, ALA 11, GLY 114, ILE 19, THR 147, GLY 20, TYR 21, PHE 36, ARG 56, LYS 57, THR 58, MET 25, GLY 23, PRO 26, LYS 24	N sp^2 – O sp^2 ILE 112<br>O sp^3 – O sp^2 ILE 19<br>O sp^3 – N sp^2 ALA 115<br>O sp^3 – N sp^2 ALA 115<br>O sp^2 – O sp^3 THR 58<br>O sp^2 – N sp^2 THR 58<br>O sp^2– N sp^2 ALA 115<br>O sp^2– N sp^2 GLU 116<br>O sp^3– O sp^2 GLU 116<br>O sp^3– N sp^3 LYS 57<br>O sp^2– N sp^3 LYS 57<br>O sp^2– N sp^2 ARG 56<br>O sp^2– N sp^2 ARG 79<br>O sp^2– N sp^2 ARG 79<br>O sp^2– N sp^2 ARG 79<br>N sp^2– N sp^2 SER 78	3.069<br>2.649<br>3.335<br>3.394<br>2.977<br>3.194<br>3,111<br>2.733<br>2.892<br>2.924<br>2.772<br>2.365<br>2.698<br>2,984<br>3.061<br>3.286
Clotrimazole	-57.87	0.10	ASP 146, THR 147, TYR 21, GLY 20, ILE 19, LYS 22, GLU 116, GLY 23, ALA 115, ILE 117, TYR 118, GLY 114, LYS 24, GLY 113, SER 61, THR 58, GLY 55, ARG 56, LYS 57		
Limonene	-41.94	0.01	GLU 116, ALA 115, ILE 117, ARG 79, SER 78, LEU 77, GLY 55, MET 54, ARG 56, LYS 57, THR 58		

Table S1 (continued)

The model generated by MOLEGRO Virtual Docker					
Co-crystallized (NDP)	-155.24	0.00	ALA 11, ALA 12, ALA 93, ALA 115, ARG 49, ARG 56, ARG 67, ARG 72, ARG 79, ARG 191, ASP 87, ASP 146, GLU 32, GLU 60, GLU 82, GLU 84, GLU 97, GLU 116, GLU 120, GLY 20, GLY 23, GLY 55, GLY 113, GLY 114, HIS 92, ILE 19, ILE 112, ILE 117, LEU 77, LEU 121, LYS 3, LYS 14, LYS 22, LYS 24, LYS 31, LYS 37, LYS 45, LYS 57, LYS 65, LYS 150, LYS 158, LYS 178, LYS 192, MET 25, SER 78, SER 80, SER 94, SER 95, THR 58, THR 147, TRP 27, TYR 21, TYR 118, VAL 10	N sp^2 – O sp^2 ILE 19	3.205
				N sp^2 – O sp^2 ALA 11	2.887
				O sp^3 – N sp^2 ALA 11	3.094
				O sp^3 – N sp^2 ALA 115	3.438
				O sp^3 – N sp^2 ALA 115	2.930
				O sp^2 – N sp^2 GLY 114	2.813
				O sp^2– O sp^3 THR 58	2.565
				O sp^2– N sp^2 THR 58	3.219
				O sp^2– N sp^2 GLU 116	3.109
				O sp^2– N sp^2 GLU 116	3.406
				O sp^2– N sp^2 ILE 117	3.303
				O sp^3– N sp^3 LYS 57	2.585
				O sp^2– N sp^2 ARG 56	3.199
				O sp^2– N sp^2 ARG 56	2.893
				O sp^2– O sp^3 SER 78	2.456
				O sp^2– N sp^2 ARG 79	2.758
				O sp^2 - O sp^2 H_2O 247	3.568
				O sp^2 - O sp^2 H_2O 352	3.163
				O sp^2 - O sp^2 H_2O 357	3.464
				O sp^2 - O sp^2 H_2O 360	2.804
Clotrimazole	-77.75	0.00	ALA 115, ASP 146, GLY 20, GLY 23, GLY 113, GLY 114, ILE 19, ILE 112, LYS 22, LYS 24, LYS 57, MET 25, SER 61, THR 58, THR 147, TYR 21, TYR 118, VAL 10	N sp^2 (N2)- O sp^3 –THR 58	2.469
Limonene	-73.71	0.00	ALA 115, ARG 56, ARG 79, GLU 116, GLY 55, GLY 113, GLY 114, ILE 117, LEU 77, LYS 57, SER 78, THR 58	-	-

Table S2. Intermolecular interactions between the compounds docked on the binding site for *S.aureus*

Ligand	Score	RMSD, Å	Group interaction	Hydrogen Bond	Bond length, Å
**Data generated by CLC Drug Discovery Workbench**					
Co-crystallized TOP	-53.12	0.35	LEU 28, ASP 27, LEU 54, VAL 31, THR 111, ALA 7, VAL 6, LEU 5, TYR 109, ILE 91, PHE 98, GLY 93, PHE 92, VAL 6, ILE 14, THR 46, LYS 45, ASN 18, SER 49, GLN 19, 1LE 50, LEU 20	N sp^2 – O sp^2 ASP 27<br>N sp^2 – O sp^2 ASP 27<br>N sp^2 – O sp^2 PHE 92<br>N sp^2 – O sp^2 LEU 5<br>O sp^3– O sp^3 –SER 49<br>O sp^3– O sp^3 –SER 49	3.143<br>3.083<br>2.589<br>3.064<br>2.987<br>2.936
Clotrimazole	-47.56	0.13	LEU 54, ILE 50, SER 49, THR 46, ASN 18, GLN 19, ILE 14, LEU 20, PRO 21, TRP 22, HIS 23, LEU 24, LEU 28	N sp^2 (N2)- O sp^3 –SER 49	3.194
Limonene	-39.63	0.02	VAL, 31 LEU 28, ASP 27, LEU 5, ALA 7, VAL 6, LEU 20, ILE 14, PHE 92, GLY 93, THR 46, GLN 95, GLY 94, THR 46, PHE 98	-	-
**Data generated by Molegro Virtual Docker**					
Co-crystallized TOP	-100.32	0.00	ALA 7, ASN 18, ASP 27, GLN 19, GLY 93, ILE 14, ILE 50, LEU 5, LEU 20, LEU 28, LEU 54, PHE 92, PHE 98, SER 49, THR 46, THR 111, VAL 6, VAL 31,	O sp^3- O sp^3 SER 49<br>N sp^2 - O sp^2 LEU 5<br>N sp^2 - O sp^2 PHE 92<br>N sp^2 - O sp^3 ASP 27<br>O sp^3 - O sp^2 –H_2O 2011<br>O sp^3 - O sp^2 –H_2O 2011<br>N sp^2 - O sp^2 –H_2O 2062<br>O sp^3 - O sp^2 –H_2O 2092	3.424<br>2.835<br>2.880<br>2.851<br>3.454<br>3.229<br>3.243<br>3.036
Clotrimazole	-59.82	0.00	ALA 7, ASN 18, ASP 27, GLN 19, ILE 50, LEU 20, LEU 28, LEU 54, PHE 92, SER 49, THR 46, VAL 6, VAL 31	N sp^2 (N2)- O sp^2 –H_2O 2092	2.998
Limonene	-50.58	0.00	ALA 7, ASP 27, ILE 50, LEU 5, LEU 20, PHE 92, PHE 98, SER 49, THR 46, VAL 6, VAL 31	-	-

Table S3 The list of intermolecular interactions between the compounds docked with 8XQ

Ligand	Score	RMSD, Å	Group interaction	Hydrogen bond	Bond length, Å
Data generated by **CLC Drug Discovery Workbench**					
Co-crystallized 8XQ	-37.48	0.03	HIS131, GLN 132, LYS 134, SER 129, ASP 133, ASP 135, LEU 128, TYR 76, MET 57, TRP 69, MET 61, ASN 120, LEU 118, ARG 210, THR 208	O sp^2– O sp^3 TYR 76<br>O sp^3– O sp^2 ASP 135<br>O sp^3– O sp^3 ASP 133<br>O sp^3– N sp^2 TRP 69<br>N sp^2– O sp^3 ASP 133	2.961<br>3.026<br>2.615<br>3.104<br>2.902
Clotrimazole	-10.06	0.01	HIS 131, SER 129, HIS 187, ARG 183, GLN 155, ASP 133, GLY 188, ILE 189, LEU 128, PHE 154, TRP 168, ALA 152, ILE 143, LEU 170, ARG 210, SER 145, THR 208, SER 147, VAL 146, ASN 206 LEU 207, ILE 119, ARG 204, LEU 218, ASN 120, MET 61, TYR 122	N sp^2 (N2)- N sp^2-GLY 188	2.866
Limonene	-45.14	0.12	GLY 157, GLN 155, PHE 154, HIS 187, TYR 186, PHE 156, PHE 185, ASP 133, LEU 184, ALA 16, SER 182, ARG 183, TRP 178, ILE 143, THR 208, ARG 210, PRO 142, GLY 179	-	-
**Data generated by MOLEGRO Virtual Docker**					
Co-crystallized 8XQ	-79.94	0.00	ARG 204, ARG 210, ASN 120, ASN 206, ASP 133, ASP 174, HIS 131, HIS 187, ILE 143, 1LE 189, LEU 128, LEU 170, PHE 154, SER 154, SER 145, TYR 208, TRP 178, TYR 122	O sp^2 - O sp^3 SER 145<br>O sp^2 - N sp^2 ARG 204<br>O sp^2 - N sp^2 ARG 204<br>O sp^2 - O sp^2 –H_2O 657<br>O sp^3 - O sp^2 –H_2O 552<br>O sp^3 - O sp^2 –H_2O 571	2.471<br>3.212<br>2.617<br>2.605<br>3.011<br>2.745
Clotrimazole	-64.85	0.00	ARG 204, ARG 210, ASN 120, ASN 206, ASP 133, GLN 155, GLY 188, HIS 131, HIS 187, ILE 143, ILE 189, LEU 118, LEU 128, LEU 170, LEU 207, PHE 154, SER 129, SER 145, SER 147, THR 208, TRP 178, TYR 122, VAL 144, VAL 146	N sp^2 (N2)- O sp^3- THR 208<br>N sp^2 (N2)- N sp^2-ARG 210<br>N sp^2 (N2)- O sp^3 –H_2O 435<br>N sp^2 (N2)- O sp^3 –H_2O 571<br>N sp^2 (N2)- O sp^3 –H_2O 552<br>N sp^2 (N1)- O sp^3 –H_2O 571	2.636<br>2.402<br>3.212<br>2.660<br>3.195<br>3.428
Limonene	-65.79	0.00	ARG 204, ARG 210, ASN 120, ASN 206, ASP 133, GLY 188, HIS 131, HIS 187, ILE 143, ILE 189, LEU 128, LEU 170,	-	-

			PHE 154, SER 145, THR 208, TRP 178, TYR 122		
